# Theoretical investigation of graphene-based photonic modulators

**DOI:** 10.1038/srep01897

**Published:** 2013-05-30

**Authors:** Jacek Gosciniak, Dawn T. H. Tan

**Affiliations:** 1 Engineering Product Development, Singapore University of Technology and Design, Singapore, 138682

## Abstract

Integration of electronics and photonics for future applications requires an efficient conversion of electrical to optical signals. The excellent electronic and photonic properties of graphene make it a suitable material for integrated systems with extremely wide operational bandwidth. In this paper, we analyze the novel geometry of modulator based on the rib photonic waveguide configuration with a double-layer graphene placed between a slab and ridge. The theoretical analysis of graphene-based electro-absorption modulator was performed showing that a 3 dB modulation with ~ 600 nm-long waveguide is possible resulting in energy per bit below 1 fJ/bit. The optical bandwidth of such modulators exceeds 12 THz with an operation speed ranging from 160 GHz to 850 GHz and limited only by graphene resistance. The performances of modulators were evaluated based on the figure of merit defined as the ratio between extinction ratio and insertion losses where it was found to exceed 220.

In recent years it has become evident that bandwidth-limited electrical interconnects can no longer meet the growing demand on data processing and telecommunication traffic. Besides the limited capacity, electrical wires suffer from large energy consumption, signal attenuation and significant operational costs as interconnects densities rise. As opposed to it, photonics enables huge amounts of data to be moved at very high speeds with extremely low power over very small optical waveguides. One of the key components of the future telecommunication networks are optical modulators which serve as the gateway from the electrical to the optical domain. These are particularly attractive for low energy transmitters because they do not have a threshold that could limit the minimum operating energy and they may be easier to integrate with the available silicon platform^1^,^2^,^3^. An optical modulator can modify the properties of light such as its phase, amplitude or polarization by thermo-optic-4, electro-optic-5, or electro-absorption modulation^6^ and they are usually based on interference (Mach-Zehnder interferometers)^7^, resonance (ring resonators)^8^ and bandgap absorption (germanium-based electro-absorption modulators)^9^. However, they suffer either from slow switching time (thermo-optic switches), narrow operating bandwidth or large footprint (electro-optic modulators). Therefore, there is a need to find new technology which will enable high operation conditions over a small active region. The unique properties of graphene such as strong coupling with light, high-speed operation, and gate-variable optical conductivity^10^ make it a very promising material for realizing novel modulators^11^,^12^. Graphene offers the highest intrinsic mobility and the largest current density of any material, as well as an extraordinary thermal conductivity. These features make graphene ideal for use in the field of nanoelectronics. A single atom layer of graphene can provide the highest saturable absorption for a given amount of material – a phenomenon which enables highly efficient electro-absorption modulators^13^, photodetectors^14^ and power monitors^15^ to be realized.

Recently, the broadband electro-absorption modulator based on interband absorption of graphene was demonstrated with an overall length of 40 μm and with a modulation depth of 0.16 dB/μm. This was achieved by placing a double-layer graphene on top of a silicon waveguide^16^. In this configuration, the effect of graphene conductivity change is not very pronounced as graphene is placed far from the electric field maximum of the propagating mode. In order to increase the effects of changes in graphene's conductivity on the propagating mode, the graphene should be placed at the maximum of the electric field^17^. It has been previously shown that plasmonics ridge waveguides^18^ are ideal candidates to realize graphene-assisted optical modulators since the electric field reaches its maximum at the interface between metal and dielectric^13^. Consequently, placing a double-layer graphene between metal and dielectric will have strong effect on the propagating mode. Another concept is based on a ridge-type photonic modulator which consists of dual graphene layers separated by thin hexagonal boron nitride (hBN) spacers placed at the center of the waveguide with maximum light intensity^11^. Although the concept is straightforward and obvious, fabrication of such structures possesses many challenges in terms of alignment, and hence, the technological imperfection might influence the resultant propagation characteristic of the mode. Compared to this configuration, the graphene-based modulator presented in this paper (Fig. 1(a)–(b)) is based on the rib waveguide platform with the graphene sheets and spacers placed between waveguide and slab. Thus, the difficulties in alignment between top and bottom ridge region are avoided. Additionally, the rib waveguide approach provides some significant advantages compared to strip waveguides as it allows for enhanced flexibility and compatibility with all processing modules such as photodiodes and multiplexers.

## Results

### Geometry and gate-variable dielectric permittivity of graphene

**Figure 1:**
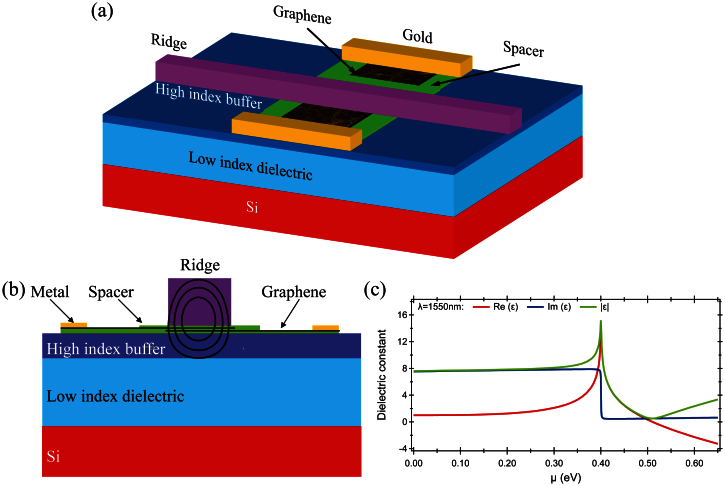
Modulator configuration. (a) Schematic representation and (b) cross-section of the rib waveguide structure with a dielectric ridge on top of a double-layer graphene and dielectric spacers on the high-index buffer dielectric (slab) with the characteristic mode field distribution (black lines). The whole structure is supported by a low-index dielectric deposited on the Si wafer. (c) Calculated dielectric constant of graphene sheet (real part, imaginary part and magnitude) as a function of chemical potential for λ = 1550 nm.

The proposed double-layer graphene optical modulator is based on the rib waveguide configuration with the Si rib waveguide deposited on the buried-oxide layer. To maximize the influence of the graphene on the modulator performances, the double-layer graphene was placed between Si layer (slab) and Si ridge (Fig. 1(b)). The double-layer graphene separated by a thin dielectric forms a simple parallel capacitor model in which the property is controlled by chemical potential μ and which can be tuned by electrical gating. Because of a synergy of the graphene properties induced by zero bandgap and symmetric valence and conduction bands, one graphene layer is doped by holes and the other by electrons at the same doping level. Thus, the applied gate voltage changes the charge-carrier density in graphene, n = α(V + V_0_), and accordingly shifts a Fermi level: 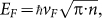
where V_0_ is the offset voltage caused by natural doping, α is estimated from a simple capacitor model (α = ε_0_ε_d_/de), ℏ is the Planck constant, v_F_ is the Fermi velocity, and n is the electron/hole doping concentration.

The gate-dependent complex dielectric function of graphene, ε(ω), was obtained from the complex optical conductivity σ(ω) = σ_1_(ω) + iσ_2_(ω) of graphene, consisting of interband and intraband contributions (Fig. 1(c)).

The dielectric constant of graphene varies very fast from ε(0.4 eV) = 13.66 + i6.71 to ε(0.7 eV) = −4.21 + i0.69 with a dip in the magnitude curve corresponding to μ = 0.51 where “dielectric graphene” is transforming to “metallic graphene” with ε(0.51 eV) = −0.50 + i0.50.

### Position of graphene sheets

**Figure 2:**
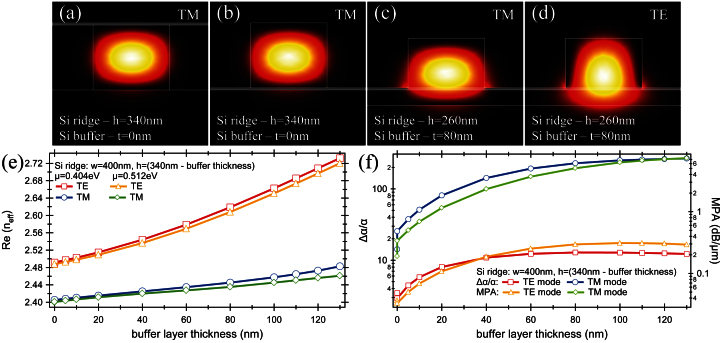
Influence of the buffer layer thickness on the modulator performances. (a)–(d) Field distribution plot of the magnitude of the power flow for considered structure with a chemical potential μ = 0.512 eV for ridge waveguide configuration (a)–(b) with a double-layer on a top of the ridge (a), and below a ridge, between SOI and Si (b). Rib waveguide configuration (c)–(d) with a double-layer placed between slab and ridge for TM (c) and TE (d) modes.

To investigate the performances of the modulator, different waveguide configurations were numerically analyzed with a double-layer graphene and dielectric spacer placed between slab and a ridge (Fig. 2). For all calculations the ridge width was kept constant at w = 400 nm and sum of the ridge height and slab height at h = 340 nm, while changing only a ratio of the ridge height to the slab height. We started our calculations with the strip waveguide (ridge height: h = 340 nm, slab height: t = 0 nm) with a double-layer graphene and spacers placed on the top of the waveguide (Fig. 2(a)). For this configuration, the modulation depth of 0.18 dB/μm and 0.037 dB/μm for TM and TE mode was calculated to have good agreement with the experimental data of 0.16 dB/μm^16^ obtained for the same configuration with the ratio of extinction ratio to insertion loss (ER-IL) 14.3 and 3.5 for TM and TE mode respectively. It should be noted that insertion losses were attributed in the paper to the propagation losses as the coupling losses from photonics waveguide to the modulator should be considered as being close to 0 dB. For the same strip waveguide configuration, an improvement was observed for graphene and spacers placed below the ridge. *i.e.,* between a buried-oxide and the Si ridge (Fig. 2(b)). For this configuration, the mode power attenuation (MPA) was calculated to be 0.34 dB/μm (TM) and 0.033 dB/μm (TE) with the ER-IL ratio of 25.5 (TM) and 2.9 (TE) respectively. Introducing a slab and reducing the ridge thickness moves the mode down, *i.e.,* the maximum of the mode electric field is closer to the graphene sheet thus strengthening the interaction between them. For only a 40 nm-thick slab and with the ridge thickness reduced from 340 nm to 300 nm, the mode attenuation reaches 2.32 dB/μm for the TM mode and 0.18 dB/μm for the TE mode what translates to a significant improvement in the ER-IL ratio to 142 and 10.9 for the TM and TE modes respectively (Fig. 2(f)). However, even better performances was obtained for 80 nm-thick slab and 260 nm-thick ridge where the MPA was 5.05 dB/μm for the TM mode and 0.29 dB/μm for the TE mode with the calculated ER-IL ratio of 230 for TM mode and 12.8 for TE mode (Fig. 2(c)–(d)). Further increasing the slab thickness, while decreasing the ridge height, causes the mode to be pushed further into the slab resulting in weaker mode field confinement as the mode is spreads into the slab. Beyond a cutoff slab thickness of 100–110 nm, a photonics mode is mostly supported in the slab layer.

It has to be emphasized that conventional GeSi electro-absorption modulators typically have ER-IL ratio which do not exceed 3.5. The optical modulators based on Si ridge waveguides integrated with graphene provide ER-IL ratios which are two orders of magnitude higher, offering a tremendous improvement over state of the art modulators.

### TM/TE mode versus chemical potential

**Figure 3:**
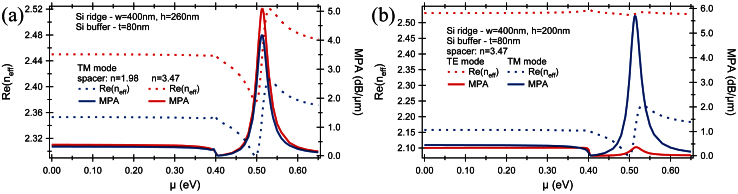
Influence of the spacer on the TE/TM modes. (a) The mode effective index (real part) and MPA of the graphene-based photonics Si rib waveguide as a function of chemical potential at λ = 1550 nm for (a) two different spacers sandwiched between Si slab (t = 80 nm) and Si ridge (w = 400 nm and h = 260 nm) and for (b) two supported modes – TM and TE for a configuration with the high-κ dielectric spacer.

Based on the results from previous section, the detailed analysis was performed for a Si rib photonics waveguide with the slab thickness of 80 nm and with a 260 nm-thick and 400 nm-wide ridge and with a double-layer graphene and spacers placed between the slab and ridge. In the first step, the change in refractive index and MPA was analyzed as a function of chemical potential for different spacer dielectrics while keeping its thickness constant at 5 nm. It has to be emphasized, that finding an appropriate dielectric spacer is another key issue which has to be addressed in obtaining efficient electro-absorption modulators. Firstly, good quality graphene sheets with high carrier mobility are formed on spacers with a small lattice mismatch with graphene. Secondly, very thin and high dielectric constant spacers are needed as it reduces the energy per bit and power consumption. Based on this, analyses were performed for two spacers with refractive indices of n = 1.98 and n = 3.47 corresponding to hexagonal boron nitride (hBN) and high-κ dielectric respectively (Fig. 3(a)).

As shown in Fig. 3(a), a similar behavior in terms of the mode effective index and MPA is observed for both dielectric spacers. However, a few changes still can be observed in terms of the mode effective index which has strong effect on realization of the electro-refractive modulators. Firstly, the higher refractive index of spacers results in a slightly larger mode effective index so for μ = 0 eV it increases from n_eff_ = 2.353 for the hBN spacer to n_eff_ = 2.450 for the high-κ dielectric spacer. Secondly, for the modulator with the low-κ dielectric spacer (hBN), the minimum mode effective index occurs at μ = 0.495 eV whereas for the high-κ dielectric spacer it shifts to μ = 0.500 eV. At the same time, the maximum mode effective index is not affected by the spacer and remains at μ = 0.530 eV. Apart from it, the difference between maximum and minimum mode effective index increases from Δn = 0.118 for the low-κ dielectric spacer (hBN) to Δn = 0.144 for the high-κ dielectric spacer. In terms of a chemical potential, the change between minimum and maximum mode effective index is Δμ = 0.035 eV for the hBN spacer and Δμ = 0.030 eV for the high-κ dielectric spacer. However, to achieve the same charge carrier mobility in graphene, a lower voltage change is required for the high-κ dielectric spacers. The required voltage change was calculated to be ΔV = 0.505 V for the hBN spacer while for the high-κ dielectric spacer it drops more than four times to ΔV = 0.141 V. Additionally, to introduce a π-phase shift between both arms of the graphene-based Mach-Zehnder interferometer, an active arm length of 6.57 μm is needed for structure with the hBN spacer and only 5.4 μm with the high-κ dielectric spacer.

For a 6.57 μm-long and 0.4 μm-wide modulator with the hBN spacer, the capacitance of the device was calculated to be 18 fF, which in tandem with the voltage change of ΔV = 0.505 V needed to ensure a full modulation results in an energy per bit on the order of 1.16 fJ/bit. Conversely, for a 5.4 μm-long and 0.4 μm-wide modulator with a high-κ dielectric it was calculated to be only E_bit_ = 0.23 fJ/bit with calculated capacitance of 46 fF and ΔV = 0.141 V.

Since the optical absorption in graphene can be controlled through electrical gating, the graphene can be used as the active medium in an optical electro-absorption modulator. Therefore, conductivity change induced by an applied gate voltage has a strong effect on the propagating mode (Fig. 3(a)). Regardless of the type of spacer material, the minimum absorption of ~ 0.02 dB/μm was observed for μ = 0.404 eV while for μ = 0.512 eV the absorption arises to the maximum of ~ 4.172 dB/μm and ~ 5.052 dB/μm for the hBN and high-κ dielectric spacers respectively. Thus, to achieve a 3 dB modulation depth a 720 nm-long waveguide is required for structure with the hBN spacer, and only a 595 nm-long waveguide for structure with the high-κ dielectric spacer. Furthermore, the efficiency of the electro-absorption modulator estimated by the figure of merit, Δα/α, shows slightly better performances with a high-κ dielectric spacer where it was calculated to be 229 compared to 224 obtained for a low-κ dielectric spacer. Apart from the optical properties, the investigated graphene-based electro-absorption modulators possess excellent electrical properties such as the energy per bit consumption. The voltage required to switch modulator from its minimum absorption state to maximum was evaluated to be ΔV = 0.453 V and ΔV = 1.394 V what corresponds to the E_bit_ = 0.26 fJ/bit for modulator with the hBN spacer and E_bit_ = 0.96 fJ/bit with the high-κ dielectric spacer respectively.

One of the ways to increase the modulator attenuation is to push mode down into a slab such that graphene resides closer to the mode field maximum. Thus, the interaction between mode field and a graphene is maximized. It can be achieved either by increasing the slab thickness or by decreasing the ridge dimensions. In our calculations the slab thickness and ridge width were kept constant while the ridge height was decreased from 260 nm to 200 nm (Fig. 3(b)). The analysis was performed for a high-κ dielectric spacer and for both supported modes – TM and TE. It was found that, as the attenuation curves for both modes exhibit similar behavior, the attenuation of the TM mode is significantly larger than that of the TE mode, and the change of chemical potential is observed to have a stronger effect on the TM mode. Most of all, for μ = 0.51 eV there is a transition from “dielectric graphene” to “metallic graphene” corresponding to a dip in the curve of dielectric constant (Fig. 1(c)). Specifically, in the absence of applied voltage, μ = 0 eV, the attenuation losses for both modes are similar with 0.34 dB/μm for the TE mode and 0.46 dB/μm for the TM mode respectively and this trend dominates up to μ ≈ 0.4 eV. This is consistent with observed trends in silicon photonics, where TM polarization is less commonly used because propagation losses tend to be higher. For μ ≈ 0.404 eV, the attenuation losses for both modes reach a minimum of 0.025 dB/μm what is manifested by decreases in the imaginary part of graphene's dielectric constant and increase in its real part (Fig. 1(c)). As μ is further increased, the losses encountered in polarizing the graphene are keep constant in low level but the polarization strength induced by a mode and described by real part of dielectric constant drops very fast what increases the MPA of both modes. However, the attenuation of the TM mode is larger than that of TE mode. At μ = 0.51 eV, the real part of dielectric constant becomes negative and the plasmonics mode associated with TM polarized light and propagating in the interface between graphene and dielectric emerges. As the negative value of the real part of dielectric constant arises, the absorption losses and MPA decrease. It should be emphasized that maximum absorption of the mode corresponds to a minimum value of the graphene dielectric constant i.e., a dip in the curve of dielectric constant magnitude^17^. This “epsilon-near-zero” effect can be seen almost in any material at its plasma frequency. However, compared to other materials, the uniqueness of graphene lies in that its plasma frequency can be tuned by electrical gating. Furthermore, the magnitude of dielectric constant varies more than 30 times between μ = 0.4 eV (maximum dielectric constant magnitude) and μ = 0.51 eV (minimum dielectric constant magnitude) what explains high modulation depth achieved with the graphene-based modulators.

### Wavelength dependence of MPA, optical bandwidth

Apart from high modulation speed, small footprint and high modulation strength (efficiency), the novel integrated modulators require large optical bandwidth for applications in on-chip optical interconnects. However, due to the poor electro-optic properties of regular materials the conventional electro-optic modulators suffer either from very large footprint or narrow bandwidth. In comparison to compound semiconductors, the ultrahigh carrier mobility and optical absorption of graphene which is independent of wavelength, enables a new optical modulators with ultra-broad optical bandwidth.

**Figure 4:**
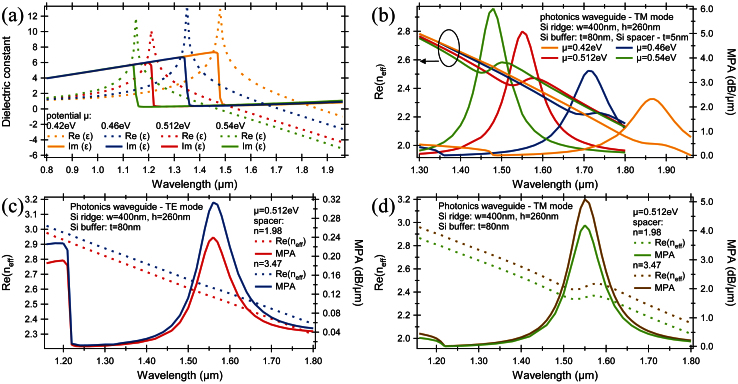
Wavelength dependence on the modulator performances. (a) Calculated dielectric constant of graphene sheet (real part and imaginary part) as a function of wavelength for different chemical potentials at λ = 1550 nm with (b) a corresponding mode effective index and MPA for TM propagating mode. (c)–(d) Mode effective index and MPA as a function of wavelength for different spacer materials for (c) TE and (d) TM modes.

To evaluate the bandwidth of the presented rib waveguide photonic modulator, the dependence of chemical potential on graphene's dielectric constant and, in consequence, on the mode effective index and MPA was studied for different wavelengths covering the entire telecommunications bandwidth. As shown in Fig. 4(a)–(b), the graphene's dielectric constant and consequently the photonics mode vary with the wavelength. Increases in the chemical potential causes a peak in the real part of dielectric constant (Fig. 4(a)), corresponding to the minimum MPA, and a dip in the magnitude of dielectric constant (transformation of “dielectric” graphene to “metallic” graphene), corresponding to the maximum MPA, move to the lower wavelengths which has direct influence on the MPA which redshift as well (Fig. 4(b)).

Thus, for a chemical potential of 0.512 eV, the dip in the magnitude of dielectric constant is observed at a wavelength of 1550 nm, what fulfill the requirements of maximum losses of the modulator. Away from the central wavelength, the MPA decreases. Consequently, the wavelength spanning from 1520 nm to 1580 nm only results in a decrease in modulation depth of ~1.5 dB/μm.

Consequently, for a lower potential, μ = 0.46 eV, the dip in the real part of the dielectric constant moves towards longer wavelengths with a maximum MPA corresponding to λ ≈ 1710 nm. Thus, for a 1 μm-long modulator, a 3 dB optical bandwidth was calculated to be 14.1 THz near 1550 nm, 12.3 THz near 1480 nm and 13.6 THz near 1710 nm for a chemical potential of 0.512 eV, 0.54 eV and 0.46 eV respectively. For a lower chemical potential, μ = 0.42 eV, a 3 dB modulation requires at least 1.5 μm-long waveguide with the optical bandwidth calculated for a 2 μm-long modulator exceeding 15.1 THz.

Apart from the chemical potential, the wavelength dependence of the MPA and mode effective index was studied for different spacer materials and for both supported modes – TE and TM (Fig. 4(c)–(d)). For a high-κ dielectric spacer and for the modulator length of 1 μm, the 5.08 dB mode attenuation can be achieved while for a low-κ dielectric spacer the same level of mode attenuation requires at least a 1.2 μm-long interaction length. The optical bandwidth for both modulators was calculated at 14.1 THz with a central wavelength at 1550 nm. Based on this it can be concluded that spacer not affect an optical bandwidth of the modulator but it impacts the modulator length (Fig. 4(d)).

For the TE supported mode (Fig. 4(c)) with a mode attenuation of 5.08 dB, the interaction length of 16.2 μm is required for structure with a high-κ dielectric spacer and 21.2 μm for a low-κ dielectric spacer. Additionally, the central wavelength corresponding to the maximum mode attenuation shifts for a TE mode from 1550 nm to 1560 nm with a 3 dB bandwidth calculated to be 16.5 THz for both dielectric spacers.

Therefore, this modulator shows broadband operation so hundreds of channels from different systems can be processed in the same device because of weak wavelength dependence.

### Modulation speed

Due to the exceptionally high carrier mobility and high saturation velocity, the operation bandwidth is not likely limited by the carrier transit time. The relaxation time is inversely proportional to the degree of crystalline disorder in the graphene so with a high quality of graphene it can operates on the timescale of picoseconds, which implies that graphene-based electronics may operate at 500 GHz. In practice, the maximum operating bandwidth is limited by the RC constant of the device.

As the calculated capacitance is very low for both dielectric spacers, the main limiting factor of the capacitive delay is graphene resistance with the graphene sheet resistance of 23.5 Ω/□ for μ = 0 eV. Thus, for modulator working in a low loss regime, *i.e.*, small losses for OFF voltage state, to obtain a 3 dB modulation it requires to introduce a modulator to a high loss regime, which is achieved by increasing the chemical potential. For a 0.72 μm-long modulator with a low-κ dielectric spacer (ε = 3.9), a 3 dB modulation is achieved with an operation speed of 0.51 THz while keeping an energy per bit at a low level of 0.96 fJ/bit. Conversely, for modulator with a high-κ dielectric spacer, the active length of around 0.595 μm is sufficient to achieve a 3 dB modulation with an operation speed of 0.16 THz and E_bit_ = 0.26 fJ/bit. Thus, it can be confirmed there is fundamental energy-speed tradeoff. Additionally, it should be noted that adaptation of longer modulators will not affect the modulation speed but will improve the modulation depth which will be however, at the cost of E_bit_. Furthermore, it can be summarized that low-κ dielectric spacers offer a higher operation speed but at the cost of energy and footprint (Fig. 3(a)).

To go beyond a ~1 THz operation speed, the modulator should be introduced in a high loss regime with OFF applied voltage state corresponding to the maximum modulation depth for μ = 0.512 eV (Fig. 3(a)). A 3 dB modulation for a 0.72 μm-long waveguide with a low-κ dielectric spacer requires significant increases in the chemical potential which can be realized by increases in the bias voltage. At the same time, with increases in the bias voltage, the graphene conductivity arises and graphene sheet resistance decreases resulting in an increase in the operation speed. Thus, for μ = 1.0 eV corresponding to voltage increases to 14 V, the bandwidth increases to 3.5 THz at the cost of the energy per bit which increases to 69 fJ/bit. Compared to it, with a high-κ dielectric spacer and for a modulator length of 0.595 μm, the bandwidth drops to 1.14 THz with E_bit_ = 18 fJ/bit for μ = 1.0 eV corresponding to voltage of 4.5 V.

## Discussion

In summary, we propose novel graphene-based photonics electro-absorption modulators based on a rib waveguide configuration. To maximize the influence of graphene on the modulator performance, the double-layer graphene was placed between a rib slab and a ridge, close to the mode field maximum. In this configuration, the conductivity of graphene was dynamically tuned by a gate voltage with a strong effect on the propagating mode. Regardless of the graphene position, it was found that TM mode gives better modulation ability than the TE mode with a modulation depth calculated to be 0.18 dB/μm and 0.037 dB/μm for TM and TE mode respectively and for the modulator configuration with graphene placed on top of the waveguide. The figure of merit in this configuration was 14.3 for the TM mode and 3.5 for TE mode. Significant improvement was observed for graphene placed between 80 μm-thick slab and 260 μm-thick ridge with a figure of merit arising to 230 for the TM mode and 12.8 for the TE mode. Additionally, the influence of the spacer on the overall performance of the modulator was considered showing that with high refractive indices spacers the modulator length can be reduced from 720 nm for the hBN spacer to 595 nm for the high-κ dielectric spacer. As optical interconnects require large optical bandwidth, the wavelength dependence of the mode attenuation was studied for different chemical potentials showing an optical bandwidth exceeding 14 THz.

Apart from the optical properties, the electrical properties of the modulator were studied in terms of the energy per bit and operation speed. It was confirmed that there is a fundamental tradeoff between energy consumption and speed. For the hBN spacer, a 3 dB modulation was achieved with an operation speed of 0.51 THz and with energy consumption of 0.96 fJ/bit whereas for a high-κ dielectric spacer it was found to be 0.16 THz and 0.26 fJ/bit respectively.

As it was shown, the presented configuration enables realization of very efficient modulators with a nanoscale footprint, small losses and with huge optical/electrical bandwidth for a future on-chip optical interconnects.

## Methods

The proposed modulator geometry was investigated using two-dimensional finite element method (FEM) simulations at the telecom wavelengths using commercial software COMSOL. The FEM is a well know technique for numerical solution of partial differential equations or integral equations, where the region of interest is subdivided into small segments and the partial differential equation is replaced with a corresponding functional. In the calculations, the refractive indexes of the Si rib, the SiO_2_ buffer and the spacers are n_1_ = 3.47, n_2_ = 1.444 and n_3_ = 1.98 for low-κ dielectric spacers and n_4_ = 3.47 for high-κ dielectric spacers, respectively

To evaluate the mode effective index and mode power attenuation of the considered structure the gate-dependent complex dielectric constant of graphene has to be calculated. The complex dielectric function ε(ω) can be obtained from the complex optical conductivity of graphene, consisting of interband and intraband contributions, 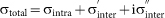

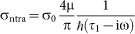

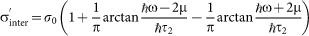

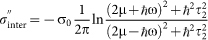
where σ_0_ = e^2^/(4ℏ) = 60.8 μS is the universal optical conductance, by using: 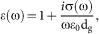
where, d_g_ = 0.7 nm is a thickness of the graphene layer.

As the complex conductivity σ(ω, μ, Γ, T) depends on the angular frequency ω, the charge particle scattering rate Γ = 1/τ with τ being the relaxation time, the chemical potential μ and temperature T^19^, the dielectric constant of the graphene layer was calculated as a function of chemical potential for λ = 1550 nm, T = 296 K (room temperature) and τ_1_ = 1.2 ps for interband conductivity and τ_2_ = 10 fs for intraband conductivity.

## Author Contributions

J.G. conceived the idea, designed the structures, performed theoretical calculations and FEM simulations and wrote the manuscript. D.T. supervised the project.
